# Development and validation of a risk score for predicting inconsistent condom use with women among men who have sex with men and women

**DOI:** 10.1186/s12889-023-15672-1

**Published:** 2023-04-21

**Authors:** Lin Chen, Tingting Jiang, Hui Wang, Hang Hong, Rui Ge, Huiling Tang, Shanling Wang, Ke Xu, Chengliang Chai, Qiaoqin Ma, Jianmin Jiang

**Affiliations:** 1 Department of HIV/AIDS and STDs Control and Prevention; Department of Zhejiang Key Lab of Vaccine, Prevention and Control of Infectious Disease, Zhejiang Provincial Center for Disease Control and Prevention, Hangzhou, People’s Republic of China; 2 Department of HIV/AIDS and STDs Control and Prevention, Ningbo Center for Disease Control and Prevention, Ningbo, People’s Republic of China; 3 Department of HIV/AIDS and STDs Control and Prevention, Jiaxing Center for Disease Control and Prevention, Zhejiang Province, Jiaxing, People’s Republic of China; 4 Department of HIV/AIDS and STDs Control and Prevention, Jinhua Center for Disease Control and Prevention,, Jinhua, People’s Republic of China; 5 Department of HIV/AIDS and STDs Control and Prevention, Taizhou Center for Disease Control and Prevention, Taizhou, People’s Republic of China; 6 Department of HIV/AIDS and STDs Control and Prevention, Hangzhou Center for Disease Control and Prevention, Hangzhou, People’s Republic of China

**Keywords:** HIV, High risk sex, Bisexual, Factors

## Abstract

**Background:**

Men who have sex with men and women (MSMW) are the most important bridge population for HIV transmission. Condom use plays an important role for HIV infection. However the predictors for condom ues with females are not well characterized.

**Methods:**

This was a cross-sectional study. Participants were enrolled by four community-based organizations (CBOs) by offline (bathrooms, bars), and online (gay applications, chat room) from April to December 2019. Electronic questionnare was fulfilled after a face-to-face training led by CBOs. We identified predictors of inconsistent condom use with females by creating a risk score based on regression coefficients. We externally validated this score via an independent cross-sectional survey conducted in Zhejiang Province in 2021. A total of 917, 615 MSMW were included in analysis in 2019 and 2021, seperately.

**Results:**

Among 917 MSMW, 73.2% reported heterosexual behavior in the prior 6 months and 38.3% reported inconsistent condom use with females (ICUF) over that time. Compared with heterosexual/unsure MSMW, bisexual MSMW reported more male and female sex partners, higher proportion of inconsistent condom use with males, less commercial sex with males (*p* < 0.05). Four risky predictors of ICUF were identified: Duration of local residence ≦6 months; more than one male partner in the prior 6 months; inconsistent condom use with males in the prior 6 months; and never heard post-exposure prophylaxis (PEP). The proportions of respondents indicating ICUF in the low- (0), medium- (2–4) and high-risk (6–20) groups (according to our risk scoring system) were 11.7% (14/120), 26.9% (96/357), and 78.1% (125/160), respectively (*P*_*trend*_ < 0.001). In the validation survey, the respective proportions of those reporting ICUF were 13.4% (15/112), 17.8% (24/185) and 87.3% (96/110) (*P*_*trend*_ < 0.001).

**Conclusions:**

We developed and validated a predictive risk score for ICUF among MSMW; four factors were identified, of which inconsistent condom use with men was the most important. Risk reduction intervention programs should focus on MSM who report inconsistent condom use with males, never heard PEP, having multiple partners and living in local less than 6 months.

## Background

Globally, men who have sex with men (MSM) continue to be disproportionately affected by human immunodeficiency virus (HIV) [1, 2]. Homosexual behavior is an important route of HIV transmission in Zhengjiang province, China which reoported an estimated 400,000 MSM [3–5]. Although the reported prevalence of HIV among men who have sex with men and women (MSMW) could be lower or higher than that among men who have sex with men only (MSMO), most studies found no difference between the two groups [6, 7]. Compared with MSMO, MSMW has led to another public health crisis regarding HIV transmission among heterosexuals. Resarches showed that the HIV prevalence among women have increased significantly, and is now as high as 28% [8, 9]; The infection of spouses and offspring caused by female infections can have a huge impact on both families and society. Furthermore, the population size of this group was not small. Studies found that 13 ~ 30% of MSM were self-reported men who have sex with men and women (MSMW) in China and the United States [6, 10–12]. Therefore, the public health risks brought by the MSMW population should also be taken seriously.

Research on MSMW has increased since 2006, when the HIV prevalence increased sharply worldwide. Recent studies have documented differences in demographic characteristics, risky sexual behavior, material abuse, physical health, HIV testing, and HIV infection among MSMW and MSMO [13–15]. The topic of condom use has always been the focus of AIDS related research for high risk population [16, 17]. However, few recent studies have focused on predictors of condom use when MSMW are with females or on interventions that target this hard-to-reach subgroup. Lack of research leads to insufficient policy strategies for promotion of condom use. China also faces the same problem, with almost no prevention and control strategies specifically targeting MSMW.

In this study, we aimed to detecting risky factors for inconsistant condom use with females among MSMW and develop a prediction model for identifying MSMW who transmit HIV to female partners. For prediction model, reference revealed that risk classification models can uncover independent predictors with high efficiency by risk score [18–20]. We tested the performance of the model via two independent studies conducted in Zhejiang Province. In addition, we explored the role of sexual orientation in this research. The study will provide recommendations for the identification of MSMW populations at risk of transmission, and provide guidance for the development of intervention strategies.

## Methods

### Study participants and design

The eligibility criteria for inclusion in this study were: (1) biological male; (2) aged 18 years or older; (3) anal or oral intercourse with a male at least once, and (4) consent to participate in the study. Those with mental retardation or inability to complet informed consent were excluded from the study.

It was a cross-sectional study. The study was complimented in four cities (Ningbo, Taizhou, Jinhua, and Jiaxing) wihich contained 60% of all MSM in Zhejiang province between April and December 2019. Four local community-based organizations (CBOs) in four cities were responsible for subjects recruitment. For convenient sampling, recruitment advertisements were posted in places where MSM gathered both offline( bathrooms, bars, parks) and online (gay applications, online chat rooms) throughout the recruiment period. Enrollment intervies were arranged in CBOs studio. At the enrollment visit, basic information was obtained and eligibility was confirmed.

Participants were asked to scan a two-dimensional code with their cellphone, and were directed to an electronic questionnaire. Electronic informed consent was obtained before the questionnaire could be completed. All participants received a face-to-face training from interviewers and then completed the questionnaire by their own in a separate room. Following the survey, participants received HIV/STI prevention risk-reduction counselling.

The sample size was caculated by PASS software (ver. 11.0; NCSS, LLC. Kaysville, UT, USA). The rate of inconsistent condom use with females, which ranges from 30 to 60%, was considered to caclulate the sample size. The minimum sample size was estimated at 631 people, with an α of 0.1 and β of 0.1.

Cellphone numbers were collected to check for duplicates, and 22 duplicate participants were identified..After removing 34 participants who did not complete the questionnair and the 22 duplicates, we enrolled 2,026 MSM. Of these, 917 were included in analysis; all self-reported that they were bisexual or heterosexual (or unsure) and had ever engaged in sex with both women and men.

Electronic questionnair was designed by National Center for AIDS/STD Control and Prevention. Information on demographic charactoristics, sexal behaivor, health seeking behavior were collected. CBOs were Responsible for conducting investigation in their studios.

### Predictor variables

The participants’ demographic characteristics were obtained, including age, registered permanent residence, length of residence in the local area, educational attainment, monthly income, marital status, and sexual orientation. Risky behavior was also assessed based on sex with males, sex with females, commercial sex with males, and condom use. Inconsistent condom use was deemed as “having sexual intercourse with no condom in the prior 6 months”. Regarding health interventions, the questions were as follows: “Have you received a message on MSM mental health via the app Blued in the last 6 months?”; “Have you ever undergone HIV testing?”; and “Have you heard about post-exposure prophylaxis (PEP)?”.

### Outcome measure

The outcome measure was inconsistent condom use with females (ICUF) in the prior 6 months, which was determined by the question “How often have you used condoms during sexual intercourse with women in the prior 6 months?”. “Used condom every time” was deemed as “consisitent condom use”. “Used condom some time or never uses” was deemed as inconsistent condom use.

### Cross-sectional validation study

External validation was performed in an independent cross-sectional study conducted from January to July 2021. This survey enrolled 1,393 MSM and 615 MSMW who met the criteria mentioned above. Of these, 407 who reported heterosexual behavior in the prior 6 months were identified. The enrollment and investigation methods were the same as in the original study of 917 MSMW conducted in the same area.

### Statistical analysis

SPSS software (ver. 20.0; SPSS Inc., Chicago, IL, USA) was used to analyze the data. Descriptive analyses were generated to describe the demographic characteristics of all subjects. The chi-square test was used to examine differences between bisexual MSMW and those who were unsure/heterosexual. A binary logistic regression model was used to evaluate risk factors and calculate odds ratios (ORs) in univariable analyses. All variables in the binary logistic regression model were also included in the multivariate model (backward logistic regression) to identified the independent factors of ICUF. Missing data were not included in the analysis, but are shown in the table and Figure. Confidence intervals (CI) and 2-sided *P*-values less than 0.05 were considered to indicate statistical significance.

We developed our risk score based on the literature [18, 19]. Points were assigned to each predictor variable based on the regression coefficients in the multivariate analysis. To validate the model, we conducted a validation study in 2021. Risk scores were generated for all subjects, who were categorized into low-, medium-, and high-risk groups accordingly. The chi-square test was used to compare inconsistent condom use among the three groups.

## Results

### Demographic characteristics of MSMW

Of 917 MSMW, more than one-third were older than 35 years (36.1%) and 27.9% had an educational level of college or above. 51.6% MSMW lived in Zhejiang and 83.9% had lived in the local area for more than 6 months. In total, 61.0% self-reported that they were bisexual, 7.2% (66) heterosexual, and 31.8% (292) unsure.

Table 1 compares the social demographics between bisexual and unsure/heterosexual MSMW. The groups differed significantly in age (χ^2^ = 39.831; *p* < 0.001), educational attainment (χ^2^ = 20.542; *p* < 0.001), and marital status (χ^2^ = 51.018; *p* < 0.001). There was no significant difference in terms of registered permanent residence, duration of local residence, or monthly income.

**Table 1:** Sociodemographic characteristics of bisexual and unsure/heterosexual MSMW of Zhejiang Province, 2019

Variables	Total (n, %)	Bisexual orientation (n, %)	Unsure /heterosexual orientation (n, %)	Chi-square/P
Age				39.831/ < 0.001
18–24	258(28.1)	132(23.6)	126(35.2)	
25–34	328(35.8)	181(32.4)	147(41.1)	
≥ 35	331(36.1)	246(44.0)	85(23.7)	
Educational attainment				20.542/ < 0.001
Junior high school and below	335(36.5)	178(31.8)	157(43.9)	
Senior high school	326(35.6)	198(35.4)	128(35.8)	
College and above	256(27.9)	183(32.7)	73(20.4)	
Registered permanent residence				2.359/0.125
Zhejiang province	473(51.6)	277(49.6)	196(54.7)	
Other provinces	444(48.4)	282(50.4)	162(45.3)	
Length of local residence (months)				0.083/0.773
≦6	143(16.1)	86(15.8)	57(16.6)	
> 6	744(83.9)	457(84.2)	287(83.4)	
missing	30			
Monthly income (RMB)				1.894/0.169
0–4999	481(56.7)	287(54.9)	194(59.7)	
≥ 5000	367(43.3)	236(45.1)	131(40.3)	
Missing	69	36	33	
Marital status				51.018/ < 0.001
Married	410(44.7)	299(53.5)	111(31.0)	
Single	467(50.9)	232(41.5)	235(65.6)	
Devoiced	40(4.4)	28(5.0)	12(3.4)	

### Status of Risky sexual behaviors

In the prior 6 months, 68.8% reported homosexual and 73.2% heterosexual behavior. Among those reporting sex with both males and females, the inconsistent condom use rates were 32.8% and 38.3%, respectively. Furthermore, 11.7% reported commercial sex with males.

Table 2 compares the risky sexual behaviors in the prior 6 months between bisexual and unsure/heterosexual MSMW. The groups differed significantly in terms of the number of male partners (χ^2^ = 69.122; *p* < 0.001), condom use during anal sex with males (χ^2^ = 13.740; *p* < 0.001), sex with female partners (χ^2^ = 10.256; *p* < 0.001), and commercial sex with males (χ^2^ = 6.646; *p* = 0.010).

**Table 2:** Risky sexual behaviors of MSMW with different sexual orientations

Variables	Total	Bisexual orientation (n, %)	Unsure /heterosexual orientation (n, %)	Chi-square/P
Number of male partners in the prior 6 months				69.122/ < 0.001
0	286(31.2)	120(21.5)	166(46.4)	
1	345(37.6)	225(40.3)	120(33.5)	
> 1	286(31.2)	214(38.3)	72(20.1)	
Condom use of anal sex with males in the prior 6 months				13.740/ < 0.001
Consistent	399(67.2)	260(62.5)	139(78.1)	
Inconsistent	195(32.8)	156(37.5)	39(21.9)	
sex with female partners in the prior 6 months				10.256/0.001
No	246(26.8)	129(23.1)	117(32.7)	
Yes	671(73.2)	430(76.9)	241(67.3)	
Condom use of anal sex with females in the prior 6 months				2.910/0.088
Consistent	414(61.7)	255(59.3)	159(66.0)	
Inconsistent	257(38.3)	175(40.7)	82(34.0)	
Commercial sex with males in the prior 6 months				6.646/0.010
No	810(88.3)	506(90.5)	304(84.9)	
Yes	107(11.7)	53(9.5)	54(15.1)	

### Develeopment of predictive risk score

Of the 671 MSMW who had sex with females in the prior 6 months, six of 10 variables were associated with ICUF on univariate analyses (Table 3).

**Table 3:** The relationship between sexual behavior, HIV intervention and inconsistent condom use with females in the prior 6 months among MSMW

Variables	Total	Inconsistent condom use n (%)	unadjusted OR	P
Age				
18–24	172	68(39.5)	1	0.065
25–34	239	78(32.6)	0.741(0.492–1.114)	
≥ 35	260	111(42.7)	1.139(0.770–1.687)	
Educational attainment				
Senior high school and above	464	157(33.8)	1	
Junior high school and below	207	100(48.3)	1.827(1.309–2.551)	0.000
Length of local residence (months)				
> 6	533	189(35.5)	1	
≦6	116	60(51.7)	1.950(1.301–2.924)	0.001
Sexual orientation				
unsure or Heterosexual	241	82(34.0)	1	
Bisexual	430	175(40.7)	1.331(0.958–1.849)	0.088
Commercial sex with males in the prior 6 months				
No	570	215(37.7)	1	
Yes	101	42(41.6)	1.175(0.764–1.808)	0.462
Number of male partners in the prior 6 months				
≦1	448	146(32.6)	1	
> 1	222	111(50.0)	2.068(1.489–2.874)	0.000
Condom use of anal sex with males in the prior 6 months				
Consistent	312	52(16.7)	1	
Inconsistent	143	123(86.0)	30.750(17.591–53.754)	0.000
No sex with male	200	70(35.0)	2.692(1.776–4.081)	0.000
Previous HIV testing				
No	487	179(36.8)	1	
Yes	184	78(42.4)	1.266(0.896–1.789)	0.181
Received mental health intervention on app “Blued” in the prior 6 months				
No	448	157(35.0)	1	
Yes	223	100(44.8)	1.507(1.086–2.091)	0.089
Ever heard PEP				
Yes	488	162(33.2)	1	
No	183	95(51.9)	2.172(1.537–3.070)	0.000

On multivariate analysis, four variables predicted ICUF: A duration of local residence ≤ 6 months (adjusted odds ratio [aOR], 1.889; 95% confidence interval [CI], 1.156–3.087), and more than one male partner in the last 6 months (aOR, 2.203; 95% CI, 1.306–3.718). Compared to consistent condom use with males, inconsistent condom use significantly increased the risk of ICUF (aOR, 27.780; 95% CI, 15.582–49.529). Compared to MSMW who had heard of PEP, those who had not were more likely to report ICUF (aOR, 1.968; 95% CI, 1.275–3.038) (Table 4).

**Table 4:** Final predictors of inconsistently condom use with females and associated risk scoring system

Variables	Regression Coefficient	P value	Odds Ratio	95%CI	Points
Length of local residence (months)(≦6)	0.636	0.011	1.889	1.156–3.087	2
Number of male partner (> 1)	0.790	0.003	2.203	1.306–3.718	2
Condom use of annual sex inconsistent	3.324	0.000	27.780	15.582–49.529	10
no sex with male	1.313	0.000	3.719	2.203–6.278	4
Ever heard PEP(No)	0.677	0.002	1.968	1.275–3.038	2

Participants with durations of local residence ≤ 6 months, with more than one male partner in the last 6 months, and who were unfamiliar with PEP were awarded 2 points on our risk score, respectively. Participants who reported inconsistent condom use with a male in the last 6 months and no sex with males were awarded 10 and 4 points (Table 4).

### Independent validation by cross-sectioanl study

**Fig. 1:**
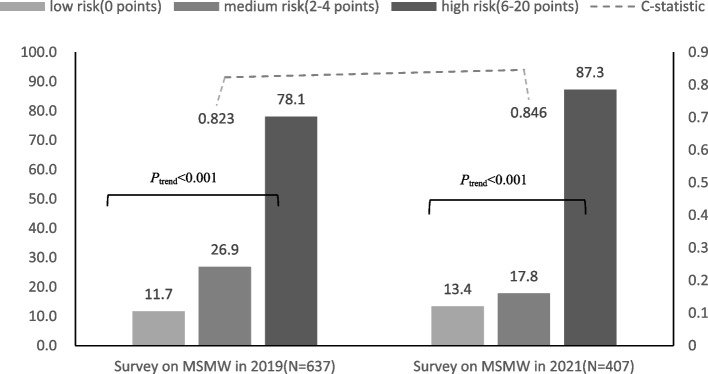
Inconsistent condom use with female among MSMW in low-, medium- and high risk classification groups. 34 participants miss information on condom use with male in survey on MSM in 2019

The proportions of respondents indicating ICUF in the low- (0), medium- (2–4) and high-risk (6–20) groups were 11.7% (14/120), 26.9% (96/357), and 78.1% (125/160), respectively (χ^2^ = 143.15, *P*_*trend*_ < 0.001). The C-statistic was 0.823 (95% CI, 0.789–0.858) (Fig. 1).

In the 2021 validation study, of the 407 MSMW who reported heterosexual behavior in the last 6 months, 135 (33.2%) reported ICUF. The proportion of ICUF was 13.4% (15/112), 17.8% (24/185) and 87.3% (96/110) in the low-, medium-, and high-risk groups, respectively (χ^2^ = 135.188, *P*_*trend*_ < 0.001). The C-statistic was 0.846 (95% CI, 0.798–0.894) (Fig. 1). 

## Discussion

Study in Tokyo showed that inconsistent condom use was reported by 37% of participants with regular male partners, 18% with casual male partners, and 20% with female partners [21]. Studies in the USA and Africa had found that MSMW are less likely to use condoms with their female than male partners [10, 22]. We found that the proportions of inconsistent condom use with females was higher than that in other reseaches and no difference of condom use was found between with males and females. Culture leads to Chinese people not being good at communicating on condom issues, especially among Chinese women. These risky behaviors need to be considered in surveillance systems to evaluate HIV transmission.

A prediction risk classification model was become popular in these years to predict the. incidence of other chronic disease and motality among HIV patients [23, 24]. In thisstudy, we applied this method to explore the predictor of condom use with females among MSMW. The risk classification model was conducted which contained 4 variables with differente risk score. Then we verified the effectivenss of this model by scoring, grouping, and conducting trend chi square tests of condom use with females on the subjects in 2019 and 2021. The results showed that length of local residence, number of male partner, condom use of annual sex and ever heard PEP were predictors of condom use with females and condom use of annual sex was the highest predictive value.

For sex with women, factors promoting condom use include disclosure of HIV serostatus to female partners, racial pride, stigma, and couple consultations [25–28]. We hypothesized that the factors affecting condom use when MSMW were with males and females would be both different and independent. However, our study showed that inconsistent condom use with males was the most important risk factor for ICUF with the highest risk score. MSM had special subculture of condom use in these years as the development of HIV prevention and their psychological characteristics [29]. MSMW is more vulnerable to condom culture than heterosexuality men which will also impacted condom use with females. Furthermore, MSMW reported insertions into males, which might be the reason for consistent condom use behavior between males and females [10]. Thus, interventions promoting condom use with males (knowledge of HIV, the attitude to condoms, self-efficacy, intention, and being skilled in the use of condoms) might prevent ICUF [30].

Living in local residence less than 6 months, having more than one male partners and never heard PEP were predictors of ICUF with the same risk score. The Province is economically well-developed in China and a large amount of funds and manpower are invested for MSM intervention. The three “90%” strategy are improved rapidly in these years with 86%, 95% and 97% in the province in 2022 [31]. MSM intervention is prioritized by CBOs online by dating apps and offline in bars, parks et al. MSM in the province can get high quality HIV/AIDS intervention, testing and treatment services. In the province, almost 40% residence are migrant from other economically underdeveloped province for work. Those living in the province less than 6 months have received far less HIV intervention services, and their awareness of safe sex is weak.

PEP, as a biological intervention, plays a very important role in HIV prevention and control [32, 33]. MSM in Chinese reported lower rate of PEP knowlege than in many countries [33]. The relationship between PEP awareness and condom use may be confusing, but it can be interpreted from a health awareness perspective. Those never hearding PEP might be kind of poplulation who are not aware of HIV infection, or have just entered the gay group who have not received enough HIV intervention. This kind of group always report risky sexual behavior, whatever with male or females. We suggest further research to detecte deep reseaon of condom use with females considering more factors.

Notably, we found no relationship between ICUF and interventions on dating app “Blued”. An digital technologies are commonly used based on apps, such as "Wechat", "Tencent", "Blued" in China [34]. App “Blued” is an dating app for finding sexual partners targating MSM and always used by govenment for HIV intervention. In fact, intervention on condom use was complimented in the recent ten years, but not special for MSMW, which could be responsible for the result. So, it should be considered the strategy targeting MSMW should be considered by govenment and make good use of internet.

Bisexual MSM are the principal bridge group for HIV transmission to females [35, 36]. In this study, compared to bisexual MSMW, heterosexual/unsure MSMW were more likely to be younger, less well-educated, and unmarried. Heterosexual/unsure MSMW reported less risky behavior with males and females in the last 6 months, more consistent condom use with males, and more commercial sex. Moreover, MSMW who are unsure of their sexual orientation also need attention; Support from family, relatives, friends, govenment was minimal and risky behavior might be high, given the traditional Chinese family culture [25, 37].

Limitations and strengths: This study had several limitations. First, the participants might not be representative of all MSM and MSMW populations in Zhejiang Province. MSMW were not randomly sampled, instead taking part on a volunteer basis (self-selection); this may have caused selection bias. Second, due to the cross-sectional design, causality could not be inferred; thus, a cohort study is needed to validate our findings. Finally, some predictors of heterosexual behavior and unprotected sex with women may have been missed. Further research should include psychological, sexual, cultural, and additional behavior indicators. As a strength of the study, to minimize bias, the introductory section of the questionnaire emphasized the need for commitment from the participant to ensure high-quality data. Also, all questionnaires were checked and revised if input errors or missing data were identified.

## Conclusion

A relatively high rate of heterosexual behavior was observed among MSM in Zhejiang Province. We identified four predictors of ICUF in MSMW, of which inconsistent condom use with men was the most important. In an independent cross-sectional study, the high-risk group had high a rate of ICUF. Risk reduction intervention programs should focus on MSM who report commercial sex with men; these individuals are likely to engage in both heterosexual behavior and unprotected sex.

## Data Availability

The data that support the study findings are available from the corresponding author upon reasonable request.

## Abbreviations

MSMW: Men who have sex with men and women
MSMO: Men who have sex with men only
ICUF: Inconsistent condom use with a females
CBOs: Community-based organizations
MSM: Men who have sex with men
ORs: Odds ratios
CI: Confidence intervals
PEP: Post-exposure prophylaxis
